# The Dentin Microbiome: A Metatranscriptomic Evaluation of Caries-Associated Bacteria

**DOI:** 10.3390/biomedicines13030583

**Published:** 2025-02-26

**Authors:** Simone G. de Oliveira, Rodrigo Jardim, Nelson Kotowski, Alberto M. R. Dávila, Hélio R. Sampaio-Filho, Karina G. S. Ruiz, Flávio H. B. Aguiar

**Affiliations:** 1 Piracicaba School of Dentistry, Campinas State University, Piracicaba 13414-903, SP, Brazil; simone.oliveira@uerj.br (S.G.d.O.); karinags@unicamp.br (K.G.S.R.); baguiar@unicamp.br (F.H.B.A.); 2 School of Dentistry, State University of Rio de Janeiro, Rio de Janeiro 13414-903, RJ, Brazil; hsampaiofilho@gmail.com; 3 Computational Biology and Systems Laboratory, Oswaldo Cruz Institute, Oswaldo Cruz Foundation, Rio de Janeiro 21040-900, RJ, Brazil; nelson.filho@ioc.fiocruz.br (N.K.); alberto.davila@fiocruz.br (A.M.R.D.)

**Keywords:** caries, RNA-Seq, mutans, lactobacillus, oxidative stress, inflammation

## Abstract

**Background/Objectives:** Dental caries remains a prevalent chronic disease globally, driven by complex interactions between the host, diet, and microbial communities. This study employs a metatranscriptomic RNA-Seq analysis to explore the functional dynamics of the dentin microbiome in both healthy and carious teeth. By examining the transcriptional activity of bacterial communities, we aimed to identify key microbial species and molecular functions associated with caries progression. **Methods:** Samples from six patients (three healthy and three decayed teeth) were analyzed using the Illumina NovaSeq 2000 platform, with data processed through the SAMSA2 pipeline for taxonomic and functional annotation. **Results:** The differential expression analysis revealed significant upregulation of *Streptococcus* and *Lactobacillus* species, including *S. mutans*, *S. sobrinus*, and *L. salivarius*, in carious samples, highlighting their roles in acid production and carbohydrate metabolism. Additionally, *Mycobacterium* species, known for their biofilm-forming capabilities and acid tolerance, were upregulated in decayed teeth. The Gene Ontology (GO) enrichment analysis identified unique molecular functions and biological processes in carious teeth, such as carbohydrate metabolism, oxidative stress response, and bacterial cell wall biogenesis, which are critical for microbial survival in acidic environments. In contrast, healthy teeth exhibited functions related to homeostasis and nutrient acquisition, reflecting a balanced microbial community. **Conclusions:** The study underscores the polymicrobial nature of dental caries, with multiple bacterial species contributing to disease progression through diverse metabolic and stress-response mechanisms. These findings provide deeper insights into the ecological shifts within the oral microbiome during caries development, emphasizing the importance of a functional metatranscriptomic analysis in understanding the pathogenesis of dental caries.

## 1. Introduction

Dental caries is a highly prevalent chronic disease worldwide, affecting people across all ages and socioeconomic groups, despite advancements in prevention and treatment. It is a complex condition resulting from interactions between the host, diet, and microbial factors [1,2,3,4]. Oral microbiome diversity plays a central role in the initiation and progression of caries. Analyzing the metatranscriptomic activity of oral bacteria can provide insights into the functional dynamics within the dental biofilm, offering a more comprehensive understanding of the ecological changes that occur during caries development and the pathogenesis of oral diseases [5].

The interaction between the microbiota of carious lesions and the host remains not fully understood. The current literature suggests that host defense mechanisms activate complex machinery involving oxidative stress pathways, an acidic environment favorable to specific bacterial species [6]. Oral bacteria exhibit regulatory and protective responses to oxidative stress, enabling their adaptation and persistence within the cariogenic environment [7].

Among the many bacterial species associated with dental caries, *Streptococcus mutans* and *Lactobacillus* spp. have been widely studied due to their prominent roles in the cariogenic process [8,9]. *Streptococcus mutans* is well-known for its ability to metabolize carbohydrates, producing acidic by-products that lower pH and lead to tooth demineralization. This bacterial species is a major contributor to biofilm formation and persistence on the tooth surface [10,11]. *Lactobacillus* spp., often detected in advanced carious lesions, are known for their acidogenicity and aciduricity, which enable them to thrive in the low-pH environment created by *S. mutans* and further promote demineralization [12].

The traditional dental caries etiological model, which centers on the acid-producing bacterium *Streptococcus mutans*, has been challenged by recent DNA- and RNA-based studies and reveals a far more diverse microbial community associated with caries lesions [13].

A metatranscriptome analysis allows for detailed investigations into genetic changes in decayed teeth and may reveal complex molecular processes that traditional methods fail to capture [5,14,15]. This approach provides a snapshot of the transcriptional activity within the oral microbiome and highlights the specific genes and pathways that are differentially expressed under various conditions [16,17,18]. Ultimately, it offers a significant advantage over traditional microbiome studies, which often focus solely on the presence and abundance of species without considering their functional roles. By investigating gene expression, we can gain deeper insights into the metabolic functions that are crucial for microbial adaptation and survival in cariogenic versus non-cariogenic environments. A metatranscriptomic analysis is a culture-independent approach that enables the study of microbial transcriptional activity directly from the environment, bypassing the need for cultivation and reducing potential biases associated with in vitro growth conditions. Additionally, with the decreasing cost of high-throughput sequencing, this method provides a cost-effective way to analyze the functional dynamics of complex microbial communities in situ, making it particularly useful for understanding the pathogenesis of dental caries.

This study aims to gain a comprehensive understanding of the microbial dynamics underlying dental caries by investigating the bacterial communities present in healthy and decayed teeth. To achieve this aim, we defined three specific objectives: to identify differentially expressed bacterial species associated with caries progression; to analyze functional profiles of microbial communities, highlighting metabolic pathways related to acid production, oxidative stress response, and bacterial adaptation to the cariogenic environment; and to compare the transcriptional activity of key cariogenic bacteria, such as *Streptococcus mutans* and *Lactobacillus* spp., to understand their role in the disease process.

## 2. Materials and Methods

### 2.1. Samples

After approval by the Research Ethics Committee of the Piracicaba Dental School (FOP/UNICAMP) under number 65630822000005418, samples were collected from 6 patients and immediately were placed in tubes with 3 mL of RNAlater™ and stored at 4 °C for up to one hour. They were then transversely sectioned using sterilized drills and handpieces while kept in the cold stabilizing solution to facilitate access to the coronal pulp. Predentin was collected by scraping the roof of the pulp chamber with a dental spoon excavator and by removing a 2 mm thick dentin slice from the area directly above the pulp chamber and immediately preserved in RNALater at −80 °C. Three samples were obtained from healthy teeth, and the other three were from decayed teeth (ICDAS 5). Inclusion criteria were the absence of antimicrobial use in the three months prior to collection, the absence of orthodontic appliances, and the absence of systemic comorbidities or other oral diseases, including periodontitis.

Total RNA was extracted from all samples using the Thermo Fisher Purelink^TM^ RNA Mini Kit (Thermo Fisher Scientific Inc, Waltham, MA, USA) according to the manufacturer’s instructions and sequenced using the Illumina NovaSeq 2000 platform (Illumina Inc., San Diego, CA, USA). The sequencing library construction involved the removal of ribosomal genes and cDNA synthesis. High-throughput RNA sequencing enabled the analysis of the gene expression profiles of microbial communities associated with healthy and decayed dentin.

### 2.2. Data Curation

Data were analyzed using the concepts of SAMSA2 (version 2.2.0) pipeline [19], a comprehensive bioinformatics tool that offers several advantages for analyzing next-generation sequencing data. SAMSA2 was employed for metatranscriptome analysis, supporting the execution of various bioinformatics programs. Since SAMSA2 was published in 2018, the pipeline was used as a guide for the analyses, with all tools and databases updated to their latest versions.

The TRIMMOMATIC (version 0.27) [20] tool was used to remove adapters from the Illumina sequencing libraries, eliminate low-quality bases (PHRED < 20), and exclude short sequences (MINLENGTH > 70). PEAR [21] was employed to assemble paired reads, enabling the unification of forward and reverse fragments of sequenced RNA and thereby increasing the sensitivity in taxonomic identification.

SORTMERNA (version 2.1) [22] was employed to remove rRNA sequences and enrich mRNA samples. This step was based on a query for ribosomal genes available within the SILVA database [23], release 138_1. Taxonomic inference was performed using the DIAMOND (version 2.1.11) program [24] against the REFSEQ database [25], considering only prokaryotic sequences.

### 2.3. Differential Expression Across Organisms

R (version 4.4.2) and Python (version 3.10) scripts from the SAMSA2 pipeline were run to generate volcano plots of organisms. Specifically, for the differential expression calculation, the parameter *p* < 0.05 was used for analyses, with |log2FC| > 1.

### 2.4. Functional Analysis with GO Enrichment

Gene Ontology [26] analyses were conducted to comprehensively identify the molecular functions and biological processes present in both healthy and decayed dental samples. The REVIGO (version 1.8.1) web server [27] was used to provide a summarized visualization and interpretation of the functional annotations obtained through the Gene Ontology enrichment analysis. Venn diagrams were generated with the InteractiVenn web tool [28], which provides an interactive visualization to compare and analyze datasets and highlight the overlapping and unique elements between them.

### 2.5. Differential Expression Between Streptococcus and Lactobacillus

Similarly, differential expression analysis across *Streptococcus* and *Lactobacillus* species and proteins was performed using R and Python scripts from the SAMSA2 pipeline, using *p* < 0.05 and |log2FC| > 0.5.

### 2.6. Statistical Analysis

The different statistical analyses were performed using SAMSA2 scripts, which use the R library DESeq2 (version 1.46.0) to calculate the differential expression and the GGPLOT2 (version 3.5.1) library to create the volcano plot.

## 3. Results

**Figure 1:**
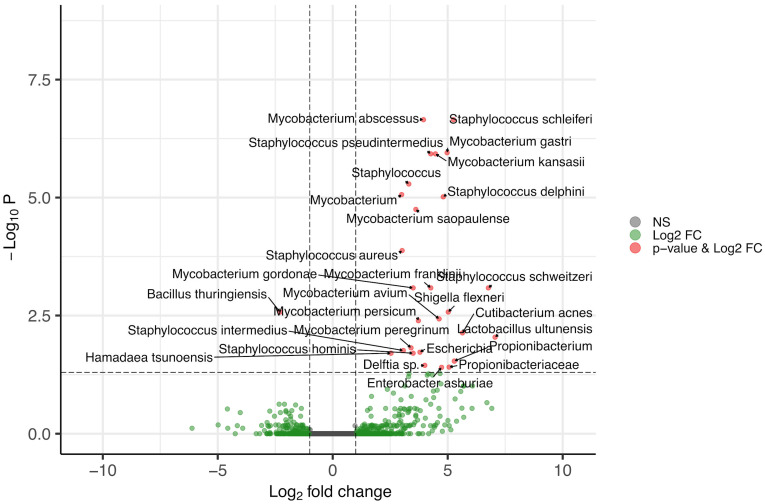
The Log2 fold-change and statistical significance of differentially expressed bacterial species. The x-axis represents Log2 fold-change in gene expression and upregulation or downregulation, while the y-axis shows the −Log10 *p*-value, where higher values denote increased statistical significance. NS denotes non-significant changes. This plot underscores the differential bacterial expression in contrasting sample conditions and provides insights into microbial activity shifts.

The differential expression analysis identified the organisms that have the highest transcriptional activity in the caries samples (Figure 1). Among the organisms that showed statistically significant differences, several genera were identified as core members of the oral microbiome, which include *Staphylococcus*, *Delftia*, *Mycobacterium*, and *Lactobacillus* [29]. Notably, the latter two genera, *Mycobacterium* and *Lactobacillus*, are of particular interest in the study of dental caries [30].

**Figure 2:**
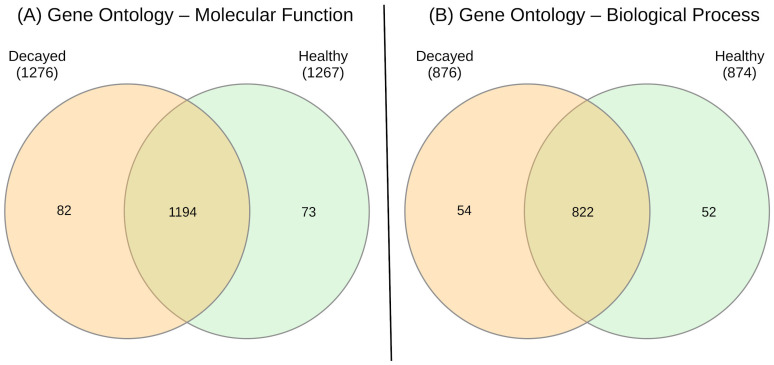
Venn diagrams depicting the overlap and unique features of Gene Ontology terms between decayed and healthy samples. (**A**) Molecular Function terms shared and unique to each condition, with substantial overlap (1194 terms) and exclusive terms for decayed (82) and healthy (73) samples. (**B**) Biological Process terms showing a similar pattern of overlap (822 terms) and unique functions for decayed (54) and healthy (52) samples. These results highlight both common and condition-specific terms associated with molecular functions and biological processes.

GO enrichment inferred molecular functions and biological processes among samples. A total of 1276 molecular functions were identified for decayed teeth and 1267 for healthy teeth, of which 1194 were common to both conditions, 82 were found only in decayed teeth and 73 only in healthy teeth samples (Figure 2A). Regarding biological processes, 876 were identified in decayed teeth and 874 in healthy teeth, of which 822 were common to both, 54 were found only in decayed teeth and 52 only in healthy teeth (Figure 2B).

**Figure 3:**
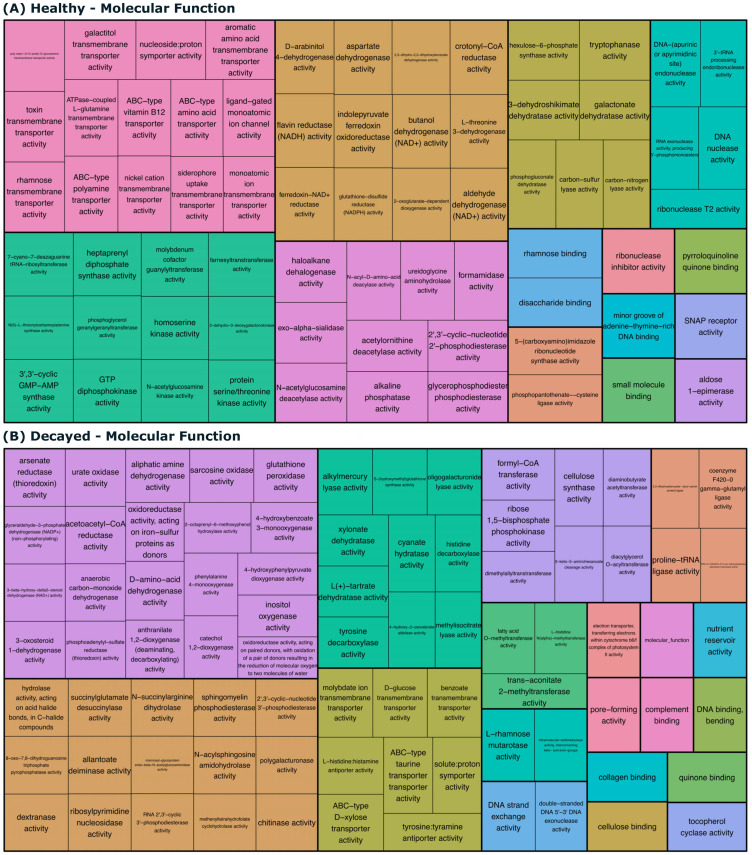
Gene Ontology (GO) Molecular Function (MF) Treemap visualization. The terms represent MF in healthy and decayed samples. (**A**,**B**) display the MF terms for healthy and decayed samples, respectively, illustrating the diversity and distribution of molecular functions identified in each condition. The size of each box reflects the relative GO term significance, with colors indicating its functional categories.

**Figure 4:**
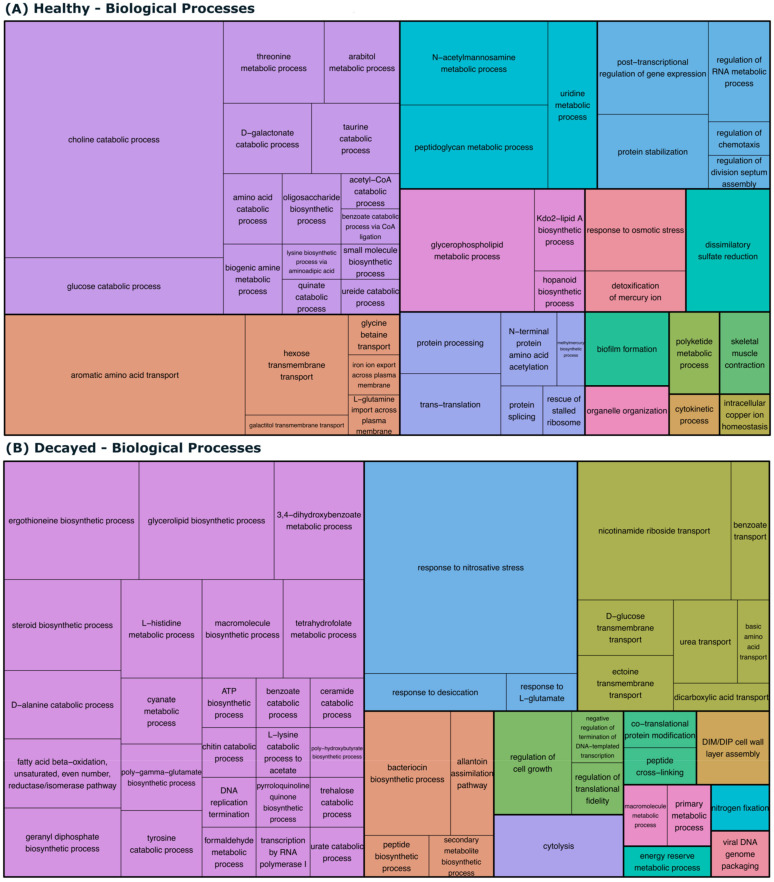
Gene Ontology (GO) Biological Processes (BP) Treemap visualization. The terms represent BP in healthy and decayed samples. (**A**,**B**) depict the BP terms for healthy and decayed samples, respectively, highlighting the biological processes prevalent in each state. The size of each box reflects the relative GO term significance, with colors indicating its functional categories.

The GO enrichment analysis also revealed that decayed teeth most-enriched biological processes and molecular functions were related to carbohydrate metabolism, oxidative stress response, and bacterial cell wall biogenesis, all of which are important for the survival and persistence of cariogenic bacteria in the dentin (Figure 3 and Figure 4).

**Figure 5:**
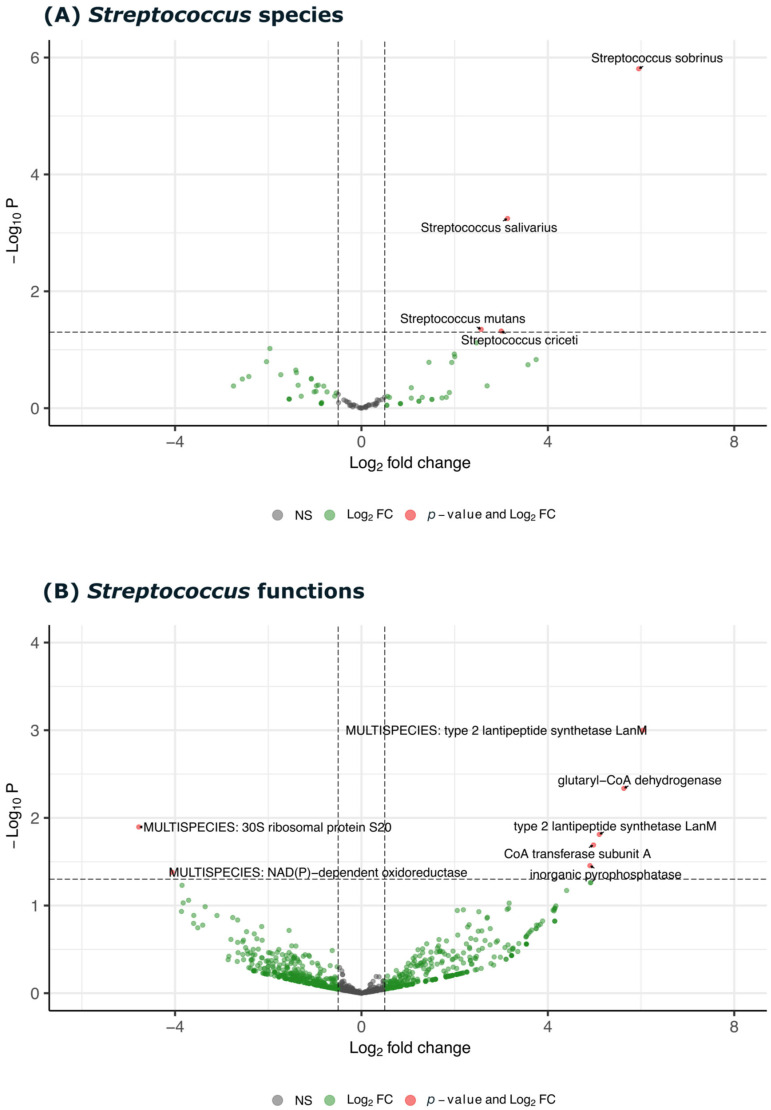
The differential expression analysis of *Streptococcus* species and their associated functions in both decayed and healthy samples. (**A**) The volcano plot showing Log2 fold change (FC) against −Log10 *p*-value for Streptococcus species, highlighting *Streptococcus sobrinus*, *Streptococcus salivarius*, *Streptococcus mutans*, and *Streptococcus criceti* as significantly differentially expressed. (**B**) The volcano plot displaying Log2 FC versus −Log10 *p*-value for specific functions associated with *Streptococcus*, including lantipeptide synthetase LanM, glutaryl-CoA dehydrogenase, and NAD(P)-dependent oxidoreductase, which are significantly upregulated or downregulated in decayed samples. Red points indicate functions or species with significant *p*-values and fold changes, green points represent Log2 FC, and gray points denote non-significant changes (NS).

Figure 5 presents two volcano plots that illustrate the differential expression of *Streptococcus* species (5A) and their proteins (5B), respectively. The first plot highlights the increased expression of *Streptococcus* species in carious samples. *S. sobrinus*, *S. mutans*, and *S. salivarius* show upregulation, suggesting their potential involvement in dental caries progression. The second plot reveals the differential expression of functional genes associated with *Streptococcus* species. The observed upregulation of proteins like lantipeptide synthetase and glutaryl CoA dehydrogenase suggests enhanced carbohydrate metabolism, acid production, and other virulence traits in the decayed samples.

**Figure 6:**
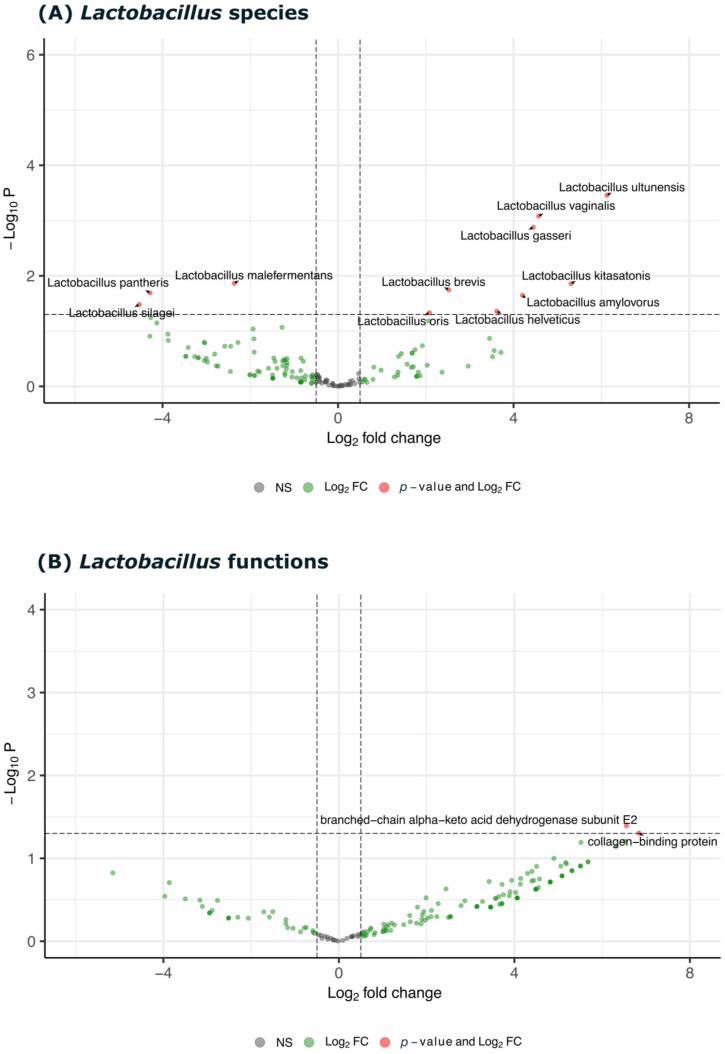
The differential expression analysis of *Lactobacillus* species and their associated functions in both decayed and healthy samples. (**A**) The volcano plot showing Log2 fold change (FC) against −Log10 *p*-value for *Lactobacillus* species, highlighting significant differentially expressed species such as *Lactobacillus ultunensis*, *Lactobacillus vaginalis*, *Lactobacillus gasseri*, and *Lactobacillus helveticus*. (**B**) The volcano plot displaying Log2 FC versus −Log10 *p*-value for specific functions associated with *Lactobacillus*, including branch-chain alpha-keto acid dehydrogenase subunit E2 and collagen-binding protein, which are significantly upregulated or downregulated in decayed samples. Red points indicate functions or species with significant *p*-values and fold changes, green points represent Log2 FC, and gray points denote non-significant changes (NS).

Similarly, the differential expression analysis of the *Lactobacillus* genus, shown in Figure 6, indicates the upregulation of several *Lactobacillus* species, including *L. salivarius*, *L. brevis*, and *L. vaginallis*, in the carious samples. The corresponding volcano plot highlights the differential expression of *Lactobacillus* proteins, revealing the upregulation of genes involved in collagen binding and acid production, which are associated with the cariogenic potential of this genus.

## 4. Discussion

This study explored the metatranscriptome of decayed teeth to evaluate the differential expression of bacterial species and the functional analysis of microbial populations associated with dental caries. The literature describes caries disease as multifactorial, with the participation of several bacterial species [5,31,32,33]. These investigation findings support the underlying comprehension that dental caries is associated with an imbalance or disruption in the microbial community structure [32,33,34].

In the analysis of differentially expressed genera, several belong to the core oral microbiome, such as *Staphylococcus*, *Delftia*, and *Mycobacterium* [29]. The presence of such genera in samples of decayed teeth may indicate their role in the dynamics of the carious process, even though they are not classically considered cariogenic. Others, such as *Shigella* and *Propioniumbacterium*, had increased expression in caries samples and corroborate previous studies that indicate their involvement in carious disease [32,33,34,35].

The cariogenic environment is characterized by a range of unique molecular functions that highlight the metabolic adaptability and resilience of the bacteria involved in dental caries. The presence of L-tartrate dehydratase and L-rhamnose mutarotase activities reflects the bacterial ability to utilize diverse carbon sources, facilitating their survival in nutrient-limited conditions [36,37]. Functions such as intramolecular oxidoreductase activity and D-amino-acid dehydrogenase suggest mechanisms for maintaining redox balance and counteracting acid stress and are important for bacterial persistence in acidic niches [38]. The benzoate transmembrane transporter and glucose transmembrane transporter activities enable efficient nutrient acquisition, while chitinase activity might contribute to microbial interactions and competition within the oral biofilm [39]. Together, these unique molecular functions underscore the presence of a bacterial community well-equipped to thrive in the cariogenic environment, leveraging metabolic diversity and stress-resistance mechanisms that likely accelerate tooth demineralization and caries progression.

In healthy teeth, the bacterial community exhibits molecular functions associated with maintaining homeostasis, acquiring nutrients, and preserving structural integrity, reflecting a less aggressive microbial environment [40]. Key functions, such as glutathione-disulfide reductase activity and ferredoxin-NAD+ reductase, suggest the presence of robust antioxidant systems that protect both the bacteria and host tissues from oxidative stress [6]. The activities of alkaline phosphatase and exo-alpha-sialidase hint at processes that support balanced nutrient cycling and cellular stability. Additionally, the transport of ions like zinc and nickel implies an adaptation to the host’s oral environment, facilitating microbial survival without contributing to tissue damage [41]. Functions such as protein serine/threonine kinase and phosphopantothenate-cysteine ligase support cellular signaling and cofactor biosynthesis and reflect an active but non-destructive bacterial community [40]. Collectively, these molecular functions represent a microbial profile compatible with oral health, characterized by metabolic processes that promote symbiosis rather than caries or tissue degradation.

Biological processes found exclusively in decayed teeth reflect the adaptation of bacteria to survive and thrive in the harsh, acidic environment characteristic of caries [42]. These microorganisms face constant stress caused by the host’s inflammatory response, as evidenced by the activation of processes such as the nitrosative stress response [43]. In addition, bacteria are known to transport and metabolize essential nutrients such as glucose, amino acids, and fatty acids, taking advantage of various energy sources available in the decayed environment [44,45].

The biosynthetic and catabolic macromolecule pathways and protein modifications indicate a bacterial effort to maintain both cellular growth and integrity [46]. At the same time, the ability to metabolize potentially toxic substances, such as formaldehyde and cyanate, reflects the resistance to adverse conditions [47]. The presence of transporters and enzymes involved in the metabolism of lipids and fatty acids is essential for the maintenance of bacterial membranes, protects against local acidity, and contributes to the dental matrix degradation [40].

The increased expression of genes involved in bacterial cell wall synthesis and adherence to the tooth surface suggests that these traits are essential for the persistence of cariogenic bacteria in the oral biofilm. These metabolic and resistance adaptations increase the ability of bacteria to survive in cavities, where the acidic environment and the accumulation of toxic compounds promote a continuous tooth structure degradation cycle. Thus, bacteria in decayed teeth employ a combination of strategies, such as stress response, nutrient transport, protein modification, and diversified metabolism, to sustain their pathogenic activity and contribute to the progression of dental caries.

Several *Mycobacterium* species are upregulated, which indicates their involvement in caries progression. The *Mycobacterium* genus has species known for their tolerance to extreme conditions, such as low pH, and their ability to form biofilms, which may confer advantages for their survival in a cariogenic environment [48]. Furthermore, the results demonstrate the involvement of *Streptococcus* and *Lactobacillus* species, commonly associated with caries. *S. sobrinus*, *S. mutans*, and *S. salivarius* showed increased gene expression in decayed samples and corroborated their connection with disease progression [32,35]. The increased expression of genes related to acid production and carbohydrate metabolism in these species suggests a central role in environmental acidification and caries initiation. Similarly, *Lactobacillus* species such as *L. salivarius*, *L. brevis*, and *L. vaginallis* demonstrated increased expression in carious samples. The high expression of collagen-binding protein by *Lactobacillus* spp. suggests that the species of this genus actively interact with the collagen-rich structure of dentin, which becomes exposed as the dentin ECM is demineralized [49]. These results indicate that the microbial consortium associated with caries progression is composed of a variety of species, and not only *Streptococcus* and *Lactobacillus*, pointing to the polymicrobial nature of the disease [13].

The study of the dentin microbiome through metatranscriptomics allowed for the identification of bacterial species and functions associated with caries disease, shedding light on its multifactorial nature. These findings underscore the complex and dynamic nature of the dentin microbiome and its involvement in the progression and development of dental caries. The interplay between various microbial entities within the oral biofilm, as well as their interactions with host-related factors, contributes to the multifaceted nature of this polymicrobial disease. The distinct clustering of caries-free and carious samples highlights the association between specific microbial community configurations and oral health versus disease states.

The study of the oral microbiota is inherently complex due to its dynamic nature and individual variability. Factors such as diet, oral hygiene habits, and host-specific behaviors play a crucial role in shaping bacterial composition throughout a person’s lifetime [50,51]. These variations can influence microbial community structure, metabolic activity, and ultimately, susceptibility to diseases like dental caries.

Microbiome and metatranscriptome studies aim to unravel these differences while identifying core microbial signatures that persist across individuals. By focusing on conserved functional interactions between bacteria and the host, these approaches help distinguish microbial adaptations associated with oral health from those linked to disease progression. Although interindividual variability remains a challenge, a metatranscriptomic analysis enables the identification of key metabolic pathways and gene expression patterns that contribute to cariogenic processes, providing a broader understanding of the polymicrobial nature of dental caries.

A metatranscriptomic analysis provides a real-time snapshot of microbial gene expression, offering insights into the metabolic activity of bacterial communities in different environments. However, it is important to recognize that mRNA abundance does not always correlate directly with protein levels or metabolic activity, as post-transcriptional and post-translational regulatory mechanisms can modulate gene expression outcomes. Factors such as mRNA stability, translation efficiency, and enzyme activity play crucial roles in determining the functional impact of differentially expressed genes [52,53].

In the context of dental caries, the upregulation of genes associated with carbohydrate metabolism, oxidative stress responses, and biofilm formation suggests an adaptive response of the microbiota to the acidic and nutrient-rich environment of decayed dentin. While these transcriptional changes provide important clues about microbial adaptation, integrating proteomic and metabolomic analyses would offer a more comprehensive view of how these molecular changes translate into functional outcomes. Future studies combining these approaches could further elucidate the metabolic shifts that sustain cariogenic bacteria and contribute to disease progression.

Despite the relevant results found in this study, the findings should be analyzed with caution regarding the bias of the study sample size. Due to the difference in the sizes of the human and bacterial genomes, the study prioritized deep sequencing, with the depletion of ribosomal transcripts, over group size.

Although a metatranscriptomic analysis captures real-time gene expression and functional activity of microbial communities, it does not directly reflect protein abundance or metabolic activity. Integrating proteomic and metabolomic data would provide a more comprehensive understanding of the biochemical processes occurring in the cariogenic environment.

The findings of this study support the polymicrobial characteristic of caries disease and highlight bacterial biological processes associated with caries. The metatranscriptomic analysis identified several molecular functions that suggest a microbial machinery adapted to oxidative stress, driven by interactions between bacteria and the host in cariogenic environments. This work was pioneering in the evaluation of dentin caries and in establishing a relationship between the microbiota of healthy and decayed teeth, contributing to the development of clinical strategies and new dental materials that minimize the progression of caries.

## Data Availability

Raw sequencing data were available in the NCBI BioProject under the code PRJNA1086832.
